# Skin Vaccination against Cervical Cancer Associated Human Papillomavirus with a Novel Micro-Projection Array in a Mouse Model

**DOI:** 10.1371/journal.pone.0013460

**Published:** 2010-10-18

**Authors:** Holly J. Corbett, Germain J. P. Fernando, Xianfeng Chen, Ian H. Frazer, Mark A. F. Kendall

**Affiliations:** 1 Delivery of Drugs and Genes Group (D2G2), Australian Institute for Bioengineering and Nanotechnology, The University of Queensland, Brisbane, Queensland, Australia; 2 Diamantina Institute, Princess Alexandra Hospital, The University of Queensland, Woolloongabba, Queensland, Australia; Massachusetts Institute of Technology, United States of America

## Abstract

**Background:**

Better delivery systems are needed for routinely used vaccines, to improve vaccine uptake. Many vaccines contain alum or alum based adjuvants. Here we investigate a novel dry-coated densely-packed micro-projection array skin patch (Nanopatch™) as an alternate delivery system to intramuscular injection for delivering an alum adjuvanted human papillomavirus (HPV) vaccine (Gardasil®) commonly used as a prophylactic vaccine against cervical cancer.

**Methodology/Principal Findings:**

Micro-projection arrays dry-coated with vaccine material (Gardasil®) delivered to C57BL/6 mouse ear skin released vaccine within 5 minutes. To assess vaccine immunogenicity, doses of corresponding to HPV-16 component of the vaccine between 0.43±0.084 ng and 300±120 ng (mean ± SD) were administered to mice at day 0 and day 14. A dose of 55±6.0 ng delivered intracutaneously by micro-projection array was sufficient to produce a maximal virus neutralizing serum antibody response at day 28 post vaccination. Neutralizing antibody titres were sustained out to 16 weeks post vaccination, and, for comparable doses of vaccine, somewhat higher titres were observed with intracutaneous patch delivery than with intramuscular delivery with the needle and syringe at this time point.

**Conclusions/Significance:**

Use of dry micro-projection arrays (Nanopatch™) has the potential to overcome the need for a vaccine cold chain for common vaccines currently delivered by needle and syringe, and to reduce risk of needle-stick injury and vaccine avoidance due to the fear of the needle especially among children.

## Introduction

Most vaccines are currently delivered by needle and syringe. However as a vaccine delivery device, the needle and syringe has many important shortcomings. These include potential transmission of blood borne diseases through needle-stick injuries [1] and needle reuse – approximately 30% of injections for the purpose of vaccination in developing nations are unsafe [2], and that needle-stick injuries cause more than 500,000 deaths per year [3]. Needle-phobia and the pain associated with an intramuscular injection are also downsides – it is estimated that needle phobia is present in at least 10% [4] of the population, or higher [5]. The muscle is also a highly inefficient site for vaccination, as it does not have a high density of antigen presenting cells. In contrast, the skin is an attractive alternative site for vaccination due to its dense network of potent antigen presenting cells (APCs) including Langerhans Cells (LCs) [6], and many sub-sets of dermal dendritic cells (dDCs) [7]. The close proximity of these cells to the skin surface means it could be possible to target them in ways which may reduce pain and potential of transmission of blood borne pathogens. While cutaneous delivery has great potential, the closest method used currently in the clinic – intradermal injection – is technically difficult, necessitating development of advanced targeting methods as reviewed in [8], [9].

**Figure 1:**
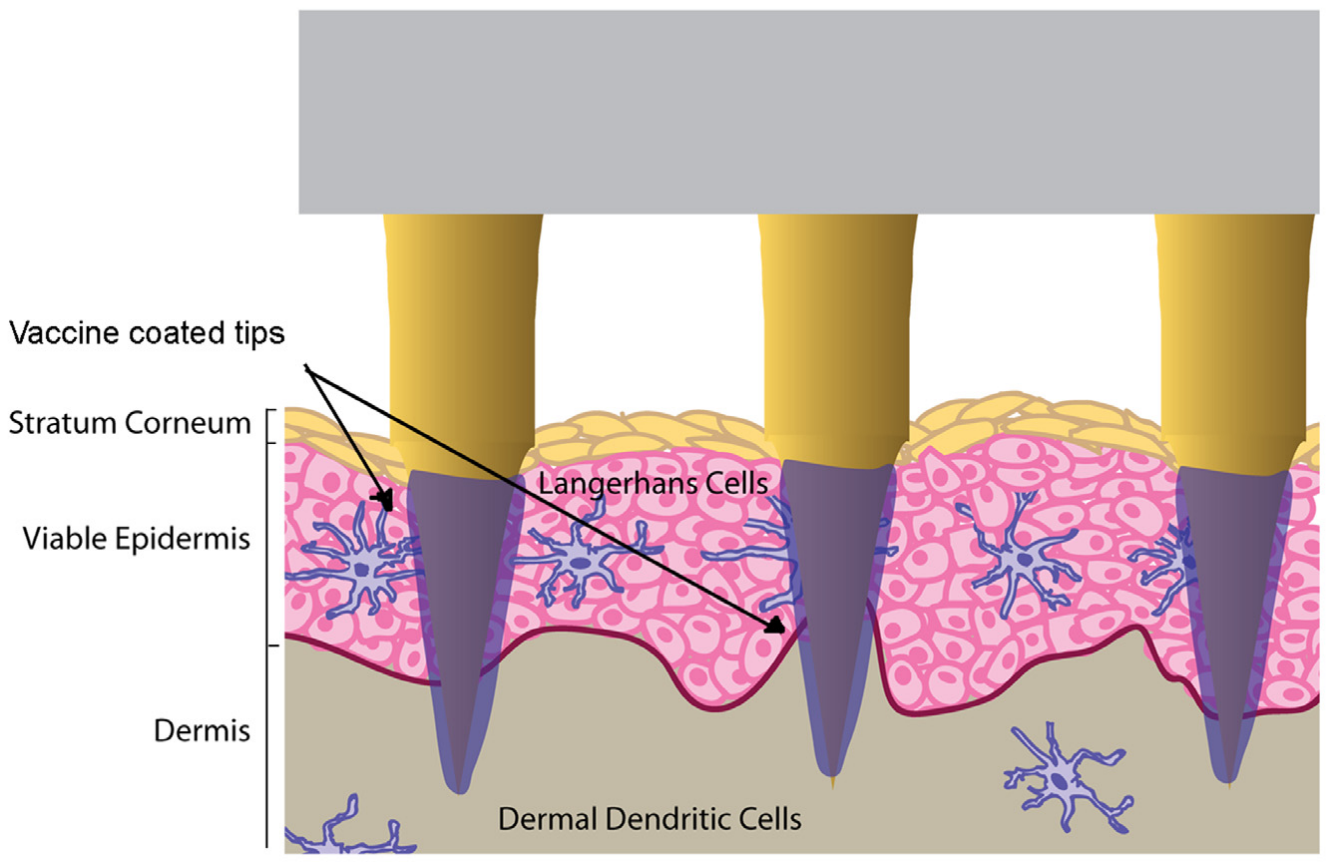
The Nanopatch™ concept. A two dimensional array of projections localizes dry coated vaccines to layers of the skin rich in immune cells. Once the vaccine hydrates, it diffuses through the viable epidermis and dermis.

In this study a novel skin patch called the Nanopatch™ is used to target these skin immune cells. The Nanopatch™ is a micro-projection array with uniquely dense projection packing (>20,000/cm^2^) and short projections (110 µm in length). This needle density was designed such that delivered vaccine has been co-localized with 50% skin immune cells – in both epidermis and dermis – upon cutaneous application without relying on diffusion (see Figure 1) [10].

Previous studies with Nanopatch™ immunization have utilized ovalbumin and split influenza vaccine as antigens without addition of an adjuvant. Crichton et. al [11] demonstrated high antibody titers after one immunization with under 2 µg via Nanopatch™ using the model antigen ovalbumin in C57BL/6 mice without a boost using 65 µm long Nanopatch projections. Fernando et. al. [10] demonstrated induction of protective levels of functional antibodies against influenza in mice with 110 µm long Nanopatch™ projections (same as used in this study) using a split virus, unadjuvanted trivalent influenza vaccine (Fluvax 2008®); with a factor of 100 in delivered dose-sparing, compared to the needle and syringe. In these previous studies, vaccines were delivered without adjuvant.

In the current study we extend to explore the utility of the Nanopatch™ in delivering an alum adjuvant. This is important, because many vaccines are adjuvanted – with Alum in the most widely used [12].

Indeed, until the recent licensure of AS04, alum was the only adjuvant to be licensed by the FDA [13]. AS04 is alum based, with the addition of a lipid based toll-like receptor 4 agonist 3-O-desacyl-4′-monophosphoryl lipid A (MPL) [14]. “Alum” is chemically either aluminum oxyhydroxide, or aluminum hydroxyphosphate. For new technologies to take advantage of currently licensed vaccines, ideally one should work with alum-adjuvanted vaccines. So far, solid formulation work has been performed with alum adjuvant for epidermal powder immunization (EPI) with hepatitis B [15], [16], [17], and diphtheria and tetanus toxoids [17]. Extensive alum gel coagulation during drying is suspected to inhibit the release of antigen such that it is not recoverable upon rehydration [16], and losses of efficacy have been reported after lyophilization or freezing [18], [19], [20]. To minimize these losses without significantly reducing the amount of alum in the total solid (<1% [18]), rapid freezing is used to prevent freeze concentration of solutes in combination with glass forming excipients.

However, in our micro-projection coating, a freeze step is not only more technically demanding, but has the potential to reduce the mechanical integrity of the coated layer. The coating must be mechanically strong enough to remain attached on needle penetration, and differing thermal expansion properties between the projections and the vaccine may cause cracking or delamination of coated layers while the device is brought to ambient temperature.

Air drying of alum containing formulations was investigated in Maa et.al.[17], and was found to cause extensive coagulation even in the presence of trehalose, and/or mannitol, and/or dextran. As current coating protocols are closer to the air drying protocols described in [17] as opposed to lyophilization or freeze drying, it is expected that vaccination with micro-projections dry coated with an alum containing formulation would result in a poor immune response. In this paper we use the soluble polymer methyl-cellulose to reduce coagulation of the gel on dehydration, and to aid in dissolution on rehydration upon application.

Our disease test case for this study is Human Papillomavirus (HPV) because – aside from the societal importance of the vaccine – assays to measure virus neutralization are well established. Antigen structure is important for raising a virus neutralizing antibody response [19], making the system sensitive to perturbations in antigen structure which may be caused by alum dehydration during dry coating.

HPV infection is causative in virtually all cases of cervical cancer – with HPV DNA present in 99.7% of cervical carcinomas [20]. Vaccination was made possible by modern recombinant protein expression systems and the discovery that expression of the L1 major capsid protein alone was sufficient to produce self-assembling virus like particles (VLPs) [21], [22]. The VLP is an empty viral particle – so it does not contain the viral genome which codes for the oncogenic proteins which cause malignancy [23], [24], [25] in a natural infection.

Commercially available vaccines Gardasil® [Merck] and Cervarix® [GlaxoSmithKline] demonstrated excellent prophylactic action in clinical trials, preventing pre-malignancies and subsequent cancers in almost 100% in the according-to-protocol analyses [26], [27], [28]. Both vaccines contain a form of alum adjuvant. Immunizations are given as three doses over the course of six months. These vaccines are prophylactic. HPV is the most common sexually transmitted infection [29], and cumulative infection incidence over 2 years post first intercourse is approximately 30%, with condom use not showing significant protective effect [30]. Immunization must occur before first intercourse to be fully protective. Many nations now have national immunization programs targeting 11 and 12-year-old girls. This has not been without issues. Mass fainting and headaches are believed to be caused by a needle phobia and mass hysteria [31], and vaccination without needles may improve vaccine uptake and acceptance.

Long term efficacy of HPV vaccines is important. While incidence varies with age, the prevalence even in women between the ages of 40 and 49 is estimated at 25.2% [32], so the potential for infection continues over a lifetime. Duration of protection is a significant factor in the cost effectiveness of HPV vaccines [33], and any second generation vaccines must demonstrate long lasting protection.

Intra-dermal injection of Canine Oral Papillomavirus (COPV) L1-glutathione-S-transferase fusion pentamers – similar to HPV pentamers – has been shown to be protective in beagles at 400 ng/dog [33]. Suzich, J.A., et al. [34] demonstrated that assembled COPV-VLP resulted in complete protection at a 50 ng dose level, and partial protection at 0.125 ng using the intra-dermal route in beagles.

The HPV VLP is assembled from 72 pentamers of the L1 protein [35], [36]. Disassembly of the VLPs can be reversibly performed *in-vitro* at high pH, low salt concentration, and with the addition of reducing agents (for example pH 8.2, 0.166M NaCl with 2 mM DTT). Dialysis against a pH 6.8 buffer with a salt concentration of 0.5–1 M results in assembly of the particles [37]. Lenz, P., et al. [38] demonstrates that HPV16L1-VLPs alone but not their constituent L1-pentamers induce maturation of dendritic cells *in-vitro*. Thönes et. al. [39] estimate that immunization with L1-pentamers also requires 20–40 fold more protein administered to obtain similar antibody response. Denatured L1 protein does not give a virus neutralizing antibody response [19], and while dry formulations can mean longer shelf life at higher temperature, a poor formulation can cause significant degradation of protein products. Suitable liquid formulations for HPV-VLP stability have been defined for both Gardasil [40] and Cervarix [41]. As the formulation has an impact on the assembly of the VLPs (and potentially the structure of the capsomeres themselves) and VLPs are more immunogenic than the capsomeres, formulation is important for the potency of a dry coated vaccine. Both vaccines a component of alum adjuvant – Gardasil contain aluminium hydroxyphosphate. To our knowledge, studies using solid formulations have not been published for HPV, or HPV adsorbed to alum.

**Figure 2:**
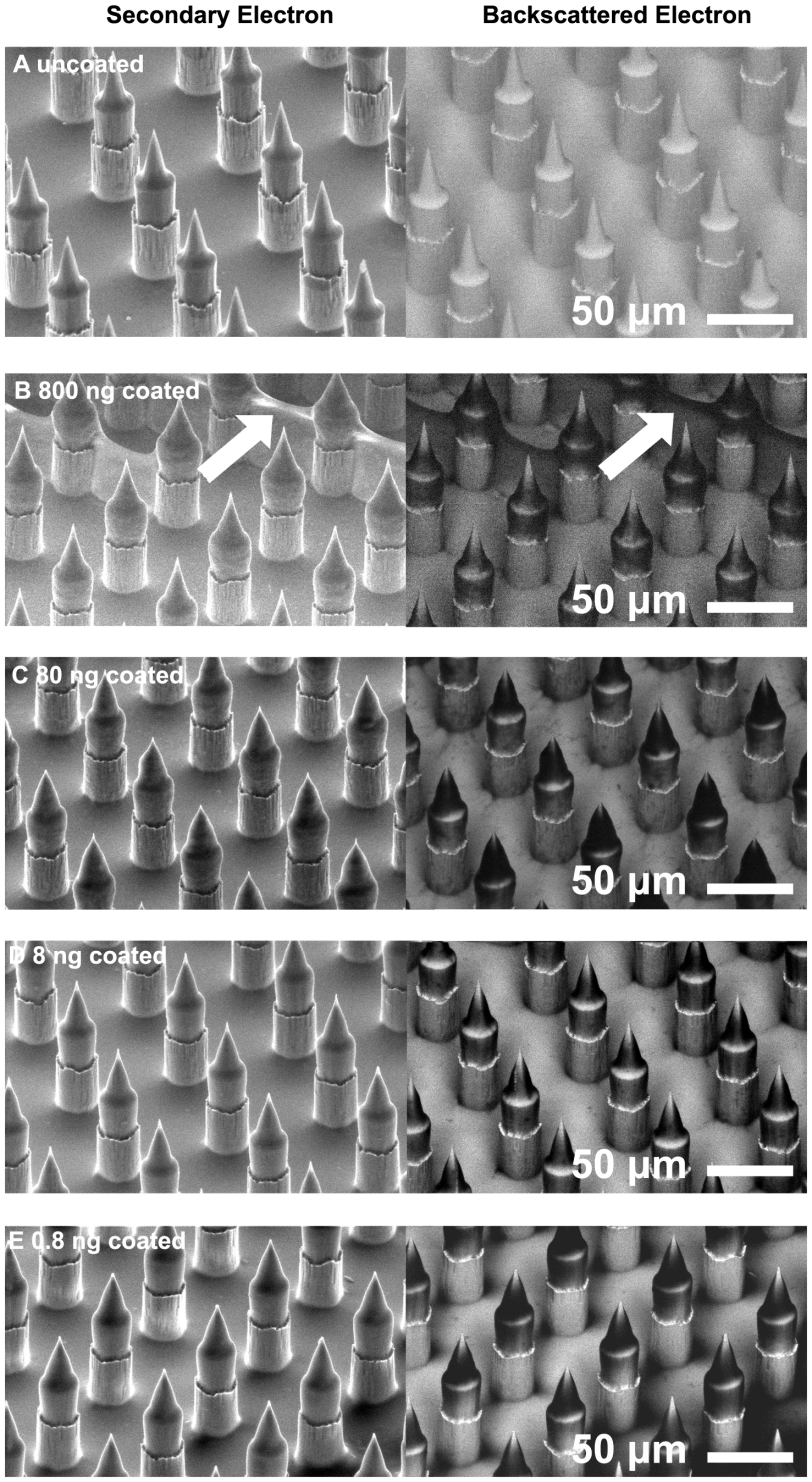
Representative scanning electron micrographs examining projection coating morphology in both secondary and backscattered electron modes. Secondary electron images show the surface morphology while backscattered electron images show composition – with low atomic mass elements giving low signal – i.e. coated area appears dark in comparison with the uncoated Nanopatch™. Micro-projections are coated at b) 800 ng, c) 80 ng, d) 8 ng and e) 0.8 ng of HPV-16 protein per patch. While some bridging is occurring in the 800 ng group (panel b – white arrow), coating is seen on the tapered portion of projections in all dose groups.

The Nanopatch™ design used in this study utilizes a 58×58 array of micro-projections 110 µm high, and 30 µm in base diameter, with a spacing of 70 µm between projection centers (see Figure 2). Vaccine is dry coated onto micro-projections as described in [42], and Nanopatches™ are applied at 2 ms^−1^. This configuration delivers material into the epidermal and dermal layers of mouse ear skin with a penetration depth of 42 µm (SD = 9.9, N = 365), as determined by imaging of fluorescent tracer in cryo-sectioned mouse ears as described in [11], but with similar projections to those utilized in [10].

In this paper, we demonstrate dry coating and release of an alum adjuvanted HPV-VLP vaccine with the Nanopatch™. We then demonstrate a subsequent immune response which is long lasting, and neutralizes the virus to levels comparable with intramuscular injection with the needle and syringe.

## Results

### Projection morphology of dry coated Nanopatches™ is suitable for needle penetration

Coated patches were examined by scanning electron microscopy (SEM) using secondary electron and backscattered modes (Figure 2) to investigate the distribution of the coated vaccine along projections. Secondary electron mode showed the surface shape of the coated needles, while backscattered electron mode was used to qualitatively confirm the thickness of the coating due to differences in the atomic mass of the gold Nanopatch™ surface and the coating. Attempts to dry coat alum adjuvant without excipient resulted in minimal vaccine coating on needle tips, and crystallization of the alum adjuvant (Figure S1). Therefore we added methyl-cellulose to stabilize the vaccine and improve coating morphology (Figure 2). A significant proportion of the coating was localized to the tapered portion of the projections. This can be seen in the secondary electron images by the difference in morphology before and after coating, and in the backscattered electron images by the dark signal on projection tips. In the 800 ng group, some bridging between projections was observed in certain areas of the Nanopatches™ (indicated by the arrow in Figure 2b) probably due to the higher concentration of vaccines than that in other groups.

### Vaccine is released from projection tips, and bulk delivery efficiency is consistent with expectations based on coating morphology and penetration data

**Figure 3:**
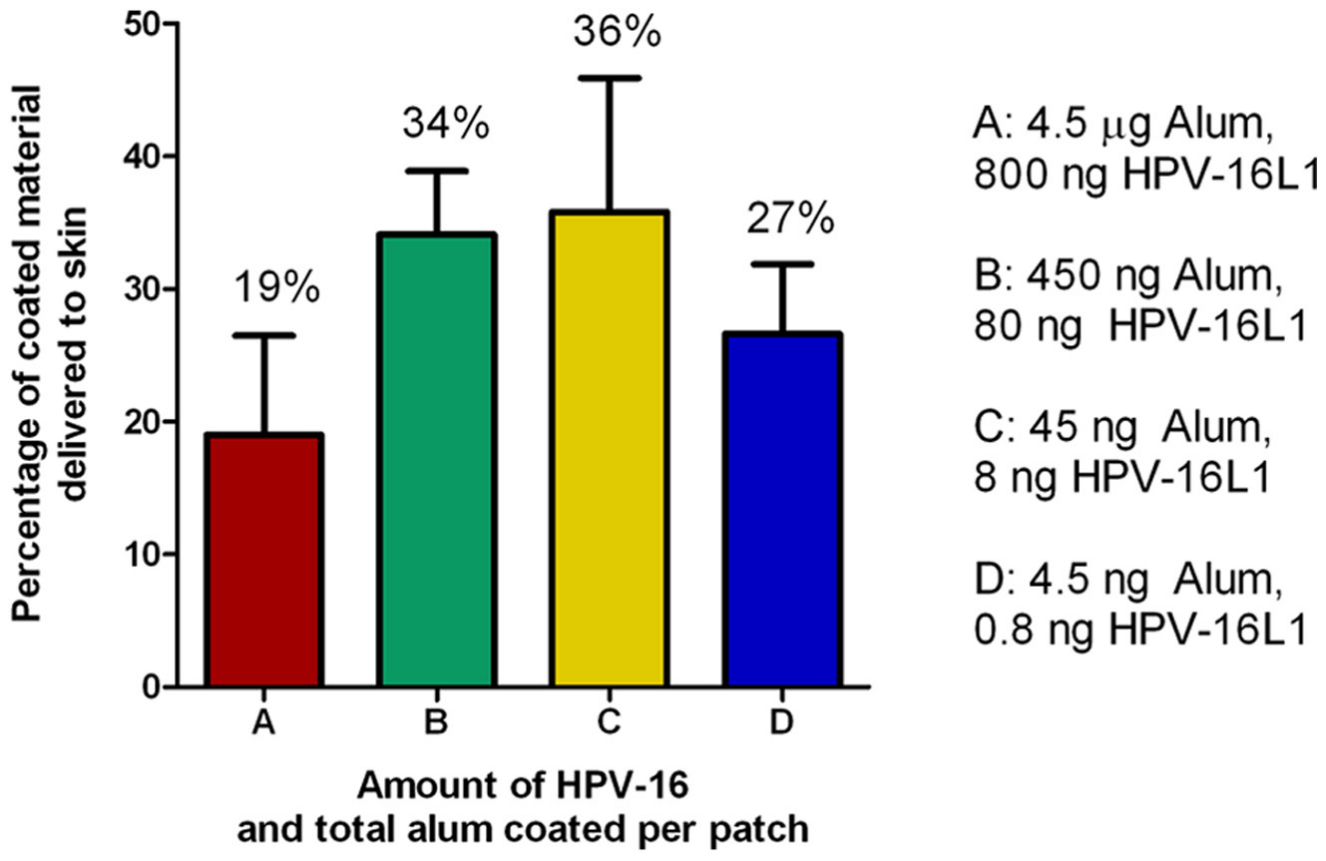
Proportion of 14C radio-labeled tracer protein coated onto the Nanopatch™ which is released into the ear skin upon application. Release was determined by mass balance, and varied between coated amounts with an average of 19%, 34%, 36%, and 27% for the formulations used for 800 ng, 80 ng, 8 ng, and 0.8 ng of coated HPV-16 per Nanopatch™.

Tenfold serial dilutions of Gardasil® were prepared and 14C labeled Ovalbumin added as a tracer to each sample, coated on Nanopatches™, and applied to the ventral earlobe of C57BL/6 mice (N = 5 per dose group) as detailed in Materials and Methods. A mass balance using the radiolabeled tracer was performed between the total coated amount, and that which was delivered into the ear skin, left on the Nanopatch™, or deposited on the surface of the skin. Efficiency of release was: 19% (SD = 7.5), 34% (SD = 4.7), 36% (SD = 10), and 27% (SD = 5.2) for 800 ng, 80 ng, 8 ng, and 0.8 ng of coated HPV-16 respectively (see Figure 3).

Delivery percentages were then used to calculate effective doses by multiplying delivery efficiency by the coated amount. Delivery amounts were estimated at 300 ng (SD = 120), 55 ng (SD = 6.0), 5.7 ng (SD = 1.6), and 0.43 ng (SD = 0.084) of HPV-16L1.

Nanopatches™ post-application were also visualized with SEM to determine whether any coating solution still remained on projections. Low molecular mass material was detected on the base in some instances, but seldom on projection tips (Figure S3)

### Nanopatch™ vaccination elicits long lasting virus neutralizing immune response, comparable with intramuscular

**Figure 4:**
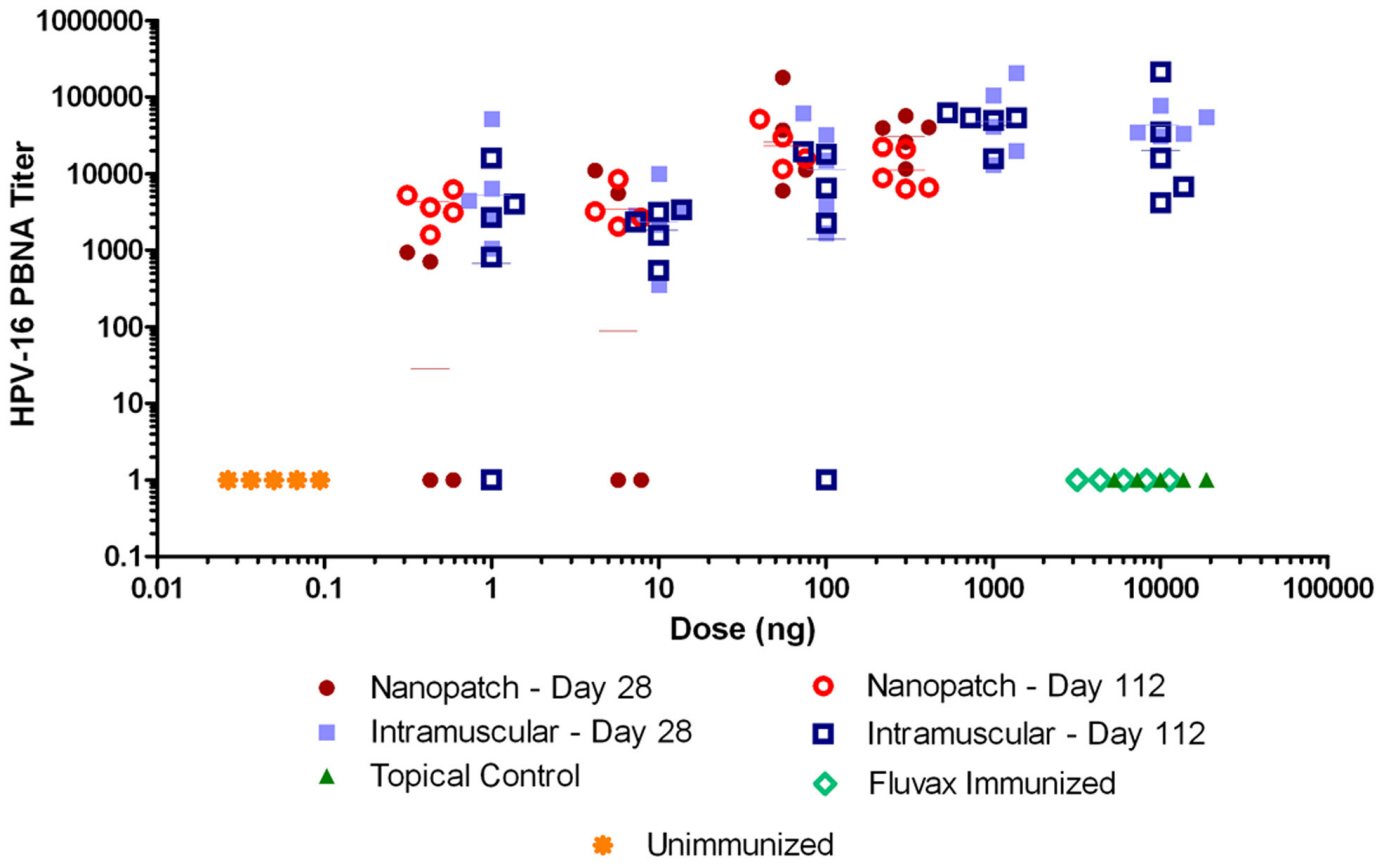
Pseudovirion neutralization assay titers at day 28 and day 112 after immunizations at day 0 and boost at day 14. Nanopatch™ doses were 300 ng (SD = 120), 55 ng (SD = 6.0), 5.7 ng (SD = 1.6) and 0.43 ng (SD = 0.084). Intramuscular doses were 10 µg, 1 µg, 100 ng, 10 ng, and 1 ng. At day 28, Nanopatch™ 300 ng and 55 ng dose groups have reached comparable virus neutralizing titers to all intramuscular injection groups (p<0.05). By day 112, all Nanopatch™ doses have reached comparable neutralizing titres against all intramuscular injection groups (p<0.05). Replicates are staggered in x axis to aid in visualization. Unimmunized plotted at a dose of 0.05 ng due to log scale.

Nanopatch™ doses were divided over each ear (i.e. one patch per ear), and administered at day 0 and again at day 14. Sera were collected at day 28 and day 112 after vaccination to examine the immediate response, and the longer term persistence of antibodies post vaccination. Sera were assayed for ability to neutralize the HPV virus with the pseudovirion-based neutralization assay (PBNA) (Figure 4). A non-inferiority analysis was used (Materials and Methods) similar to previous Human Papillomavirus immunization comparison studies [43].

At day 28 post-immunization, titers in the 300±120 ng and 55±6.0 ng dose Nanopatch™ groups were statistically non-inferior to all intramuscular doses (p<0.001) with geometric mean titer (GMT) of 30500 and 26000 respectively (Figure 4). In each of the 5.7±1.6 ng and 0.43±0.084 ng dose level Nanopatch™ groups, two mice did not seroconvert.

Neutralizing antibodies were titered at day 112 post-immunization (Figure 4) to establish long-term efficacy. At day 112 post-immunization, all Nanopatch™ groups showed 100% sero-conversion. At day 112, all Nanopatch™ doses were statistically non-inferior to all intramuscular doses (p<0.05).

When comparing the response at day 112 and day 28 within each group (Figure 4), the day 112 titer did not meet non-inferiority criteria against the day 28 titer in both the 100 ng and 1 ng intramuscular groups (ratio of titres: 100 ng 90% CI (0.40, 1.2), and 1 ng 90% CI (0.39,1.2)). In contrast, within group Nanopatch™ titer comparisons showed titres at day 112 were non-inferior in all cases, and superior at the 0.43±0.084 ng dose level, with the day 112 titer being 1.2–5.8 times higher than the day 28 titer (p<0.05) at the same dose.

## Discussion

This paper demonstrates effective coating and release of an alum containing vaccine Gardasil® from a novel micro-projection array patch (Nanopatch™) designed to deliver vaccine directly to about 50% of the abundant skin antigen presenting cells [10], for efficient induction of a virus neutralizing antibody response.

Release efficiency varied between dose groups. It is predicted that this is primarily dependant on the coating morphology. This is supported by our theoretical delivery expectations (Figure S2). Our predicted releases (Figure S2) as compared with measured release were most accurate within the lowest dose groups. We over-predicted the release in the highest dose groups. This discrepancy is likely to be due to bridging between projections and excessive coating on the base of Nanopatches™ (Figure 2b) leading to wastage of coating solution. Since coating on the base and bridging projections were not accounted for in calculations, where these amounts are significant, it would result in an overestimate of delivered amount as the theoretical calculations were only based upon coating solution on the projections. This bridging will likely be alleviated by the addition of a surfactant in later formulations. These data, and the post application images demonstrate that the release from the projections is complete, and thus with advances in coating technology to isolate coating solely to portions of the projections which penetrate skin, complete release 100% efficiency is possible with these formulations.

Fast release is likely to aid in ensuring consistent vaccine dose is delivered, as it is easier to ensure compliance with the immunization protocol. An observation period of 15–20 minutes following a standard intramuscular immunization is generally recommended as most anaphylactic shock will occur within this period [44]. Release of the dry coated vaccine from the Nanopatch™ occurred within five minutes – thereby easily meeting observation period guidelines.

To demonstrate that virus neutralizing antibodies were raised, serum was screened by the Pseudovirion-Based Neutralization Assay (PBNA) – considered the “gold standard” in the WHO guidelines for HPV vaccines [45], [46]. This assay measures the effectiveness of antibodies or other compounds in blocking the entry of the HPV virus into 293TT cells [47]. This is significant, as protection is believed to be a result of antibody mediated virus neutralization [48], [49]. The method also removes the bias of using the same material for both immunization and immunological assay which occurs when ELISA is used. Assay signal is only generated by intact pseudovirions that mimic the native virus, thus any immune response raised against contaminants or denatured protein will not impact the results of the neutralization assay. Nanopatch™ immunization with 0.43±0.084 ng of HPV-16 resulted in virus neutralizing response at day 112 after vaccination. This implies that the dry coated L1 protein maintains some native virus-like structure. We speculate that some degree of disassembly of VLP into pentamers may have occurred. This assertion is based on our similar studies using viral vectors (unpublished data). Owing to the increased immunogenicity of VLPs in comparison with pentamers, if disassembly into pentamers is currently occurring during dry-coating, a great increase in activity is likely through reformulation to further preserve VLP structure.

While early results showed only 50% sero-conversion in the 5.7±1.6 ng and 0.43±0.084 ng Nanopatch™ groups, advantages become clearer at the terminal bleed when all mice have sero-converted. At the lower doses intramuscular began to falter, with the titers of two mice dropping below the detection limit. It is possible that Nanopatch™ immunization, with alum containing vaccines, has slower release kinetics as compared with intramuscular administration – owing to the different administration site and a greater depot effect from dehydrated alum. While extremely low doses of HPV-VLP appear immunogenic intramuscularly in mice, doses for humans in the approved vaccines are in the 20–40 µg range per subtype. Slow release kinetics may favorably alter immunological outcomes and assist in achieving optimal activation of APCs without the need to physically apply more doses. Further work is required to investigate this.

Previous studies of intradermal immunization with COPV [33], [34] utilized an ELISA assay so a direct comparison with our results is not possible. Suzich et al [34] studied the intradermal delivery of assembled COPV-VLPs and obtained partial protection at sub-nanogram dose level. By comparison, a later study by Yuan, et. al. [33] with intradermal administration of COPV-L1-GST fusion protein (unable to assemble into VLPs) showed no detectable response by ELISA below a 400 ng dose. This work focused on assembly incompetent pentamers as an inexpensive alternative to VLPs. In both instances, partial protection was noted against challenge even when antibodies were not detected. Nanopatch™ immunization with split virion influenza vaccine gives superior dose reduction against intramuscular [10] as compared with what has been demonstrated in intradermal administration [50]. If disassembly of HPV-VLPs is occurring in our Nanopatch™ dry coating, the closest effective dose comparison is with the work of Yuan et al. This would suggest that Nanopatch™ immunization may give increased immunogenicity in a direct comparison with intradermal administration (and thus presumably intramuscular administration) of L1-pentamers. However, as the currently licensed vaccines are VLP based, reformulation to preserve VLP integrity will be the focus of future work.

Our release assays measure the proportion of coating which is released into the skin – they do not measure what proportion of antigen is effectively released from the alum, nor its conformation on release. If the alum gel is coagulated, in accordance with previous studies [17], antigen may not escape the collapsed alum matrix – thus reducing the effective dose. This effect may in fact negate any adjuvant benefits the alum otherwise provides, though further study is required to determine the extent to which the alum matrix impairs release, and its impact. Future work will investigate protein structure and coagulation of the alum as well as excipients to reduce it. As HPV-L1-VLPs are highly immunogenic even without adjuvant, formulations without alum will also be investigated. We speculate that with enhanced formulations, Nanopatch™ immunization may eventually mean lower doses of alum adjuvanted VLP can be used to elicit a lasting immune response in humans.

Future work will focus on improving dose sparing in Nanopatch™ immunization, and investigating thermostability. To this end, dry coated VLP integrity, exploring unadjuvanted VLPs and improving formulations will be explored.

## Materials and Methods

### Ethics Statement

All animal experiments were conducted according to the University of Queensland animal ethics regulations.

### Nanopatch™ Fabrication

Nanopatches™ were fabricated by Deep Reactive Ion Etching according to patent [51] in the Rutherford Appleton Laboratory, Oxford, UK. Nanopatches™ were sputter coated with gold to a thickness of 100 nm. Uniform morphology was confirmed by Scanning Electron Microscopy (SEM) on a Philips XL30. Samples were tilted at 45° to confirm appropriate projection profile.

### Nanopatch™ Coating

Gardasil® (Merck, USA) was centrifuged at 5000 g for 15 minutes to pellet the alum with the adsorbed virus like particles. The supernatant was removed and the pellet re-suspended in methylcellulose to a final concentration of 10 µg/µL methylcellulose, and 100 ng/µL of HPV-16-VLP. Ten-fold serial dilutions were prepared in 10 µg/µL methylcellulose to formulate different doses. Methocel® 60 HG - Methyl-cellulose (cat # 646555) was purchased from Sigma Aldrich (Castle Hill, NSW, Australia).

Nanopatches™ with 110 µm long needles were coated by pipetting 8 µL of coating solution onto the surface and dry coating via nitrogen jet as previously described [42]. Appropriate coating morphology was confirmed by SEM.

### Nanopatch™ Immunization

Four groups (N = 5) of female C57BL/6 mice at 6 wks old were anesthetized with Ketamil® and Xylasil®. Mice were then patched once in each ear on the ventral side using a spring application device to apply the patch at 2.0 ms^−1^ - a velocity found to give maximal needle penetration without damaging tissue (data not shown). Doses stated in this paper refer to total HPV-16 dose for each immunization distributed over two Nanopatches™, and mice were immunized at day 0 and boosted at day 14. Patches were held in situ for 5 minutes. This study was carried out in strict accordance with the recommendations in the Guide for the Care and Use of Laboratory Animals of the National Health and Medical Research Council (Australia). The protocol was approved by the Committee on the Ethics of Animal Experiments of the University of Queensland (Permit Number AIBN/020/10 (NF)). All efforts were made to minimize suffering.

### Intramuscular injections

Gardasil® doses were concentrated by centrifugation at 5000 g for 15 minutes. Excess supernatant was removed and pellet was re-suspended in the appropriate amount of supernatant to give an HPV-16 VLP concentration of 0.2 µg/µL. Four 10-fold serial dilutions were prepared into the excess supernatant.

Five groups (N = 5) of C57BL/6 mice at 6 wks old were immunized intramuscularly at day 0 and boosted at day 14 with 25 uL delivered approximately 2 mm into each caudal muscle of the hind leg with each group receiving 10 µg, 1 µg, 100 ng, 10 ng, or 1 ng of HPV-16 per mouse per immunization.

### Control Application

Topical control: One group (N = 5) of C57BL/6 mice at 6 wks old were anesthetized with ketamil and xylasil. Mice were restrained with ventral ear surface flat, and 125 µL of Gardasil® (containing 10 µg of HPV-16) was applied topically for five minutes.

Fluvax immunized control: One group (N = 5) of C57BL/6 mice at 6 wks old were anesthetized with Ketamil® and Xylasil®, and immunized intramuscularly with 6 µg of Fluvax 2008® (CSL Ltd., Melbourne, Australia) in a volume of 30 µL.

Unimmunized control: One group (N = 5) of C57BL/6 mice at 6 wks were anesthetized with Ketamil® and Xylasil® and sacrificed immediately upon cessation of reflexes by cardiac puncture.

### Sera collection

Blood samples were obtained at day 28 and 112 after vaccinations by retro-orbital bleeds, and serum separated. Sera were stored at −20°C until analysis.

### Scanning Electron Microscopy (SEM)

Samples were examined in secondary electron and back scattered electron modes in a Philips XL30. Samples were tilted to 45° to enable visualization of the needles, and imaged at an accelerating voltage of 20 kV.

### Predicted delivery image analysis

The canny edge detection method was used in MATLAB® 2010a (The Mathsworks™) to generate the outline of a silhouette of projection shapes on both coated and released Nanopatches™. Silhouettes were filled in and checked manually as binary images, with coated projections at a grey level of 0 and released projections at a grey level of 255, and images were overlaid to generate a binary image of expected coating shape. For each projection, a region-of-interest was created based on the proportion of the projection expected to penetrate skin from fluorescent dye studies [11] and ratios of amount of coating on each projection tip as compared with the whole projection were taken.

### Radiometric delivery analysis

1.5 ml vials of Gardasil® were concentrated to 330 µL via centrifugation, and tenfold serial dilutions were made in saline – final alum concentrations – 1 µg/µL, 100 ng/µL, 10 ng/µL, 1 ng/µL, and 0.1 ng/µL all in a volume of 300 µL. 80 nCi of 14C Radio-labeled ovalbumin tracer (American Radiolabeled Chemicals Cat# ARC 0431) was added to each vial, and incubated for one hour at room temperature. Binding efficiency of tracer to the alum within 30 minutes was 95%. The mixtures were then centrifuged at 5000 g for 15 minutes, the supernatants collected in vials for scintillation counting, and the pellets re-suspended in 10 mg/mL methylcellulose to a final volume of 54 µL. Nanopatches™ were coated with the radio-labeled formulation as described above, and applied to mouse ears (N = 5/dose) as described for Nanopatch™ immunization. Ear surfaces were swabbed to remove vaccine left on the surface of the ear and not delivered into the cell layers below. Ears were excised and solubilized in Soluene (Perkin Elmer) for 4 hours at 60°C. Swabs, ears and used Nanopatches™ were placed in scintillation vials and 10 mL of Hionic Fluor (Perkin Elmer) was added to each vial. Samples were counted for two minutes per sample in a liquid scintillation counter.

### Pseudovirion-Based Neutralization Assay (PBNA)

Pseudovirion based Neutralization assay was performed as previously described [52]. Briefly 100 µL of 293TT (generously provided by John Schiller, NIH, USA) cells were plated at 3×10^5^ cells/mL in 96-well plates and allowed to attach. Serum was diluted in three-fold serial dilutions from 1∶100 to 1∶1968300, mixed with type 16 pseudovirions expressing secreted alkaline phosphatase (SEAP) (plasmids p16shell and pYSEAP generously provided by John Schiller, NIH, USA), and pre-incubated on ice for 1 hour before addition to cell layers. In plate controls were five wells of cells without pseudovirion applied, and five wells of cells with pseudovirion applied at the same concentration as in wells incubated with serum. Plates were incubated for 3 days at 37°C, and SEAP expression was quantified using the colorimetric approach. Plates were read at 405 nm absorbance, and data was normalized between cells only and pseudovirion only wells. Log transformed data was fitted with the four parameter dose-response model (GraphPad™ Prism v5.03 ). Neutralization titer was calculated as the theoretical dilution of serum which gives 50% reduction in SEAP expression (EC50 from the model). Non sero-converted samples were assigned an arbitrary titer of 1.

### Non-inferiority analysis

Two-sided 90% confidence intervals (CI) of the anti-HPV-16 titer ratios (Nanopatch™ divided by comparison group) were calculated on the log_10_ transformation of the ratio between titers under comparison using the Fieller's theorem [53]. If the lower tail of the CI was greater than 0.5, non-inferiority was concluded. If the lower tail of the CI was greater than 1.0, superiority was concluded.

## Supporting Information

Figure S1 Coating without excipient. Representative secondary electron (left - S.E) and backscattered electron (right - B) SEM images of a NanopatchTM coated without adding the polymer methylcellulose. Crystallization of the coating is evident, and backscattered electron imaging confirms that coating is localized towards the base of the micro-projections and the NanopatchTM.
(0.57 MB TIF)

Figure S2 Theoretical delivered amounts compared with measured. Theoretical (marked with asterisks) and actual proportion (solid bars) of 14C radio-labeled tracer protein coated onto the NanopatchTM which is released into the ear skin upon application. Theoretical released amount was determined by image analysis of coated NanopatchesTM, and varied between coated amounts with estimates of 35%, 48%, 36%, and 36% for the formulations used for 800 ng, 80 ng, 8 ng and 0.8 ng of coated HPV-16 per NanopatchTM.
(2.02 MB TIF)

Figure S3 NanopatchesTM after five minute application to mouse ear skin. Secondary and backscattered electron scanning electron micrographs of Nanopatches™ after 5 minute application to the ear skin. Backscattered electron images show the atomic mass of compounds imaged - thus darker areas represent lower atomic number elements - such as the coating solution. It can be seen that coating is no longer on projections as in figure 3. Low atomic mass material on the base of projections as seen in panels d and b may be either coating solution or biological matter post-application.
(6.53 MB TIF)
