# Supplementary figures and images for: Skin Vaccination against Cervical Cancer Associated Human Papillomavirus with a Novel Micro-Projection Array in a Mouse Model
Source: PLoS One. 2010 Oct 18;5(10):e13460. doi: 10.1371/journal.pone.0013460 (PMC2956639; doi:10.1371/journal.pone.0013460)

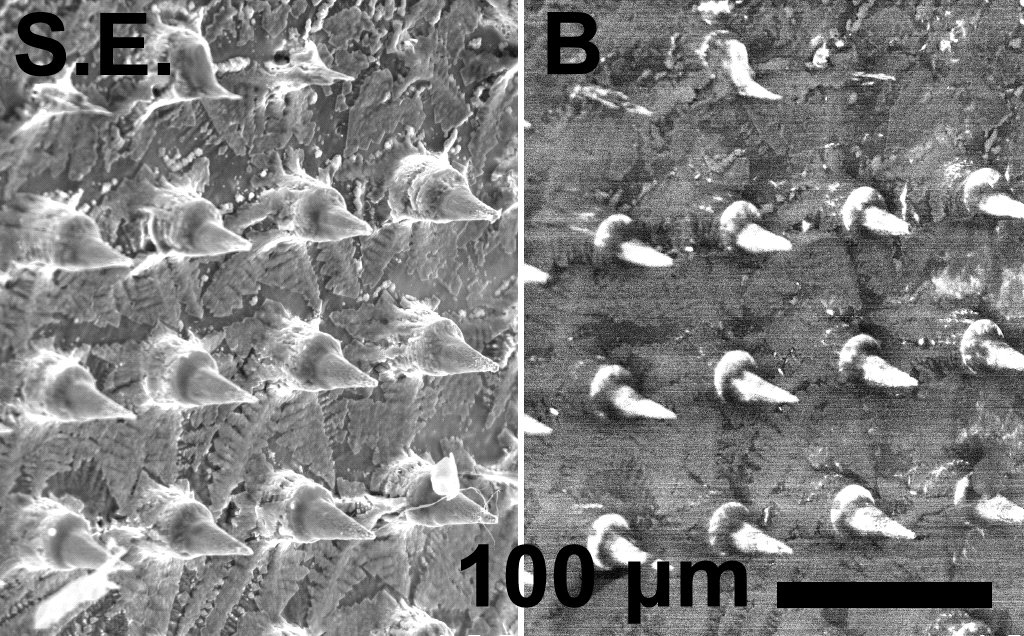

Supplement: Figure S1 — Coating without excipient. Representative secondary electron (left - S.E) and backscattered electron (right - B) SEM images of a NanopatchTM coated without adding the polymer methylcellulose. Crystallization of the coating is evident, and backscattered electron imaging confirms that coating is localized towards the base of the micro-projections and the NanopatchTM. (0.57 MB TIF) [file pone.0013460.s001.tif]

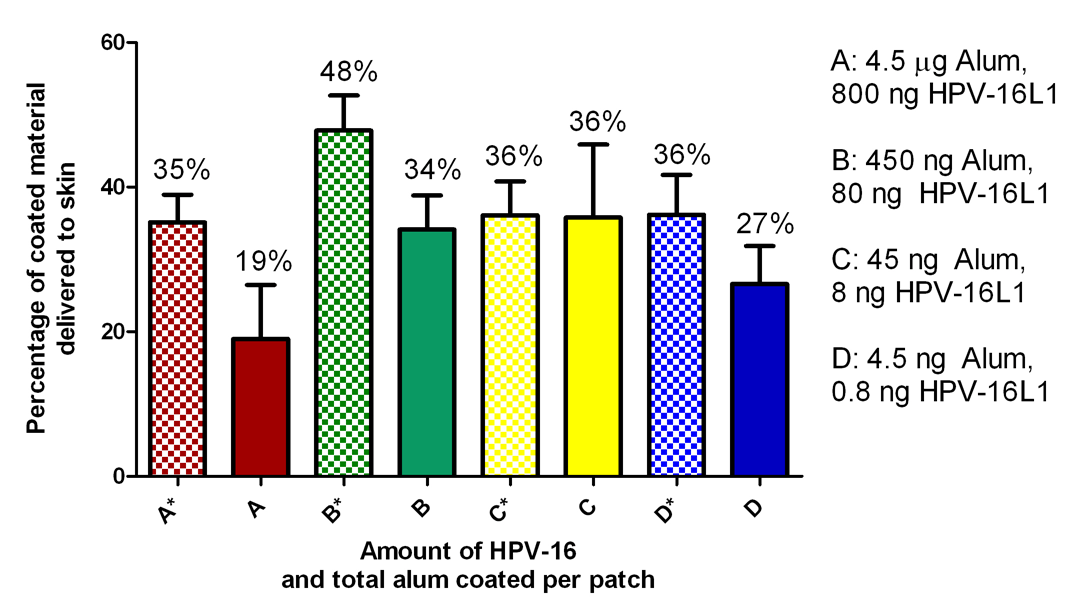

Supplement: Figure S2 — Theoretical delivered amounts compared with measured. Theoretical (marked with asterisks) and actual proportion (solid bars) of 14C radio-labeled tracer protein coated onto the NanopatchTM which is released into the ear skin upon application. Theoretical released amount was determined by image analysis of coated NanopatchesTM, and varied between coated amounts with estimates of 35%, 48%, 36%, and 36% for the formulations used for 800 ng, 80 ng, 8 ng and 0.8 ng of coated HPV-16 per NanopatchTM. (2.02 MB TIF) [file pone.0013460.s002.tif]

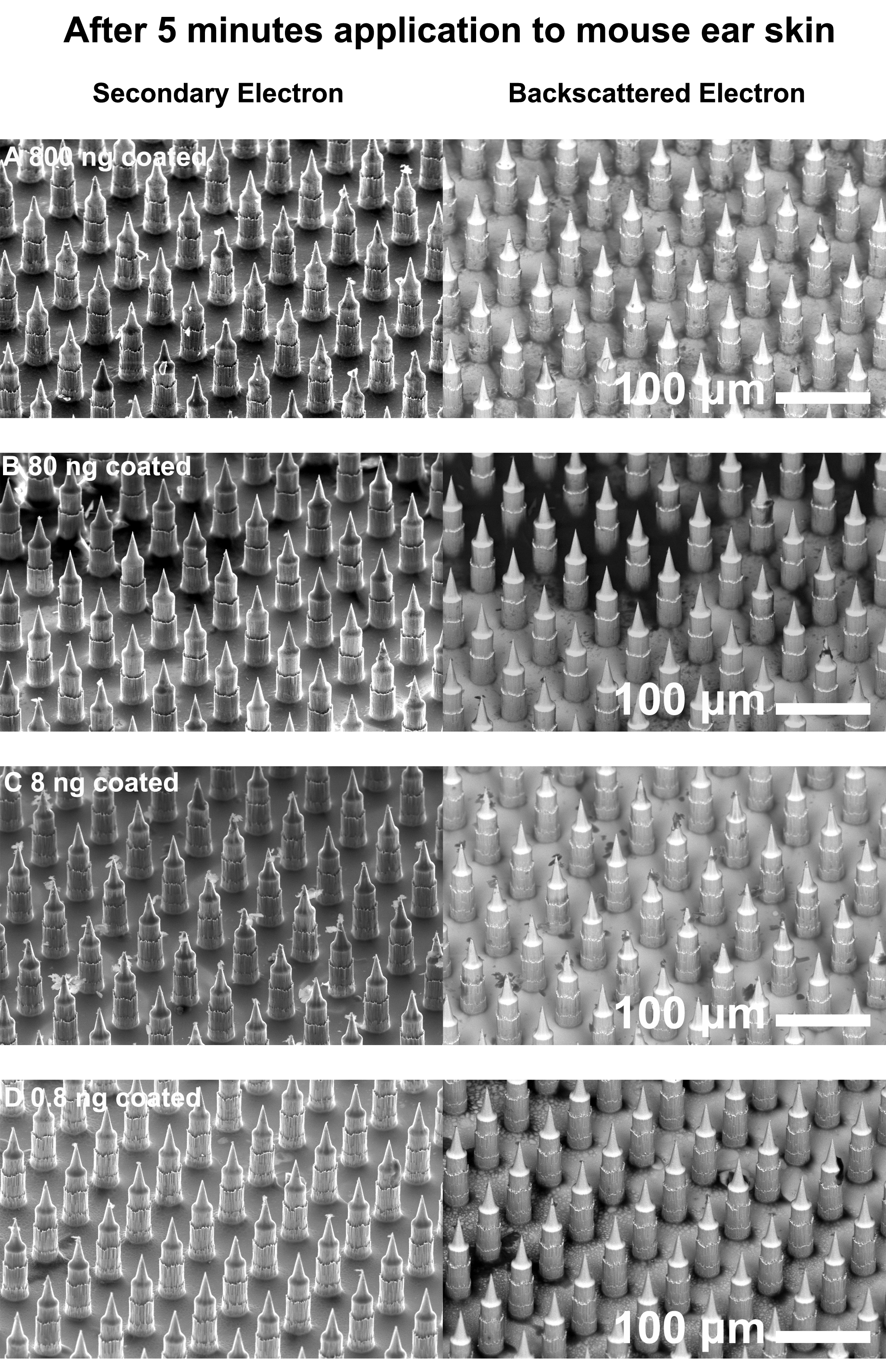

Supplement: Figure S3 — NanopatchesTM after five minute application to mouse ear skin. Secondary and backscattered electron scanning electron micrographs of Nanopatches™ after 5 minute application to the ear skin. Backscattered electron images show the atomic mass of compounds imaged - thus darker areas represent lower atomic number elements - such as the coating solution. It can be seen that coating is no longer on projections as in figure 3. Low atomic mass material on the base of projections as seen in panels d and b may be either coating solution or biological matter post-application. (6.53 MB TIF) [file pone.0013460.s003.tif]
